# Estrogen Deficiency Induces Mitochondrial Damage Prior to Emergence of Cognitive Deficits in a Postmenopausal Mouse Model

**DOI:** 10.3389/fnagi.2021.713819

**Published:** 2021-07-15

**Authors:** Wei Zhao, Yue Hou, Xinxin Song, Lei Wang, Fangfang Zhang, Hanting Zhang, Haiyang Yu, Yanmeng Zhou

**Affiliations:** ^1^Institute of Pharmacology, Shandong First Medical University and Shandong Academy of Medical Sciences, Taian, China; ^2^Departments of Neuroscience and Behavioral Medicine and Psychiatry, Rockefeller Neurosciences Institute, West Virginia University Health Sciences Center, Morgantown, WV, United States

**Keywords:** estrogen, ovariectomy, cognitive function, mitochondrial biogenesis, mitochondrial dynamics, mitophagy, hippocampus

## Abstract

**Background**: Estrogen deficiency contributes to the development of Alzheimer’s disease (AD) in menopausal women. In the current study, we examined the impact of estrogen deficiency on mitochondrial function and cognition using a postmenopausal mouse model.

**Methods**: Bilateral ovariectomy was conducted in adult females C57BL/6J. Cognitive function was examined using the Morris water maze (MWM) test at 2 weeks, 1, 2, and 3 months after ovariectomy. Neurodegeneration was assessed using an immunofluorescence assay of microtubule-associated protein 2 (MAP2) in the hippocampus and immunoblotting against postsynaptic density-95 (PSD95). Mitochondrial function in the hippocampus was assessed using immunoblotting for NDUFB8, SDHB, UQCRC2, MTCO1, and ATP5A1. Mitochondrial biogenesis was examined using immunoblotting for PGC-1α, NRF1, and mtTFA. Mitochondrion fission was assessed with immunoblotting for Drp1, whereas mitochondrion fusion was analyzed with immunoblotting for OPA1 and Mfn2. Mitophagy was examined with immunoblotting for PINK1 and LC3B. Mice receiving sham surgery were used as controls.

**Results**: Ovariectomy resulted in significant learning and memory deficits in the MWM test at 3 months, but not at any earlier time points. At 2 weeks after ovariectomy, levels of Drp1 phosphorylated at Ser637 decreased in the hippocampus. At 1 month after ovariectomy, hippocampal levels of NDUFB8, SDHB, PGC-1α, mtTFA, OPA1, and Mfn2 were significantly reduced. At 2 months after ovariectomy, hippocampal levels of MAP2, PSD95, MTCO1, NRF1, and Pink1 were also reduced. At 3 months, levels of LC3B-II were reduced.

**Conclusions**: The cognitive decline associated with estrogen deficiency is preceded by mitochondrial dysfunction, abnormal mitochondrial biogenesis, irregular mitochondrial dynamics, and decreased mitophagy. Thus, mitochondrial damage may contribute to cognitive impairment associated with estrogen deficiency.

## Introduction

Alzheimer’s disease (AD) is a neurodegenerative disease affecting over 6 million individuals globally, which is projected to increase to 14 million by 2060 (Matthews et al., 2019). AD is characterized by progressive memory loss, cognitive impairment, and abnormal behavior (Selkoe, 2001). Commonly recognized histopathological features of AD include intracellular neurofibrillary tangles (NFTs), extracellular amyloid-β (Aβ) deposition, neuron loss, synaptic damage, as well as changes in mitochondrial structure and function (Bloom, 2014; Fu et al., 2017; Wang et al., 2017; Reddy et al., 2018).

Many studies have indicated that abnormal mitochondrial bioenergetics is an important pathological feature of several neurodegenerative diseases (Swerdlow, 2018; Fišar et al., 2019). In order to maintain their function, neurons require substantial energy. As the main energy producer, mitochondria are crucial to the survival and proper function of neurons. Mitochondria produce ATP to maintain a variety of basic synaptic functions including ion gradients across the cell membrane, the release and circulation of synaptic vesicles, and the plasticity of synapses (Attwell and Laughlin, 2001; Li et al., 2004; Lee and Peng, 2008; Sun et al., 2013; Rangaraju et al., 2014). Mitochondria are highly dynamic organelles that constantly fluctuate with the changing metabolic and physiological needs of cells. The morphology and number of mitochondria are controlled through fusion and fission (Karbowski and Youle, 2003; Santel and Frank, 2008).

Since mitochondria play a crucial role in neuronal health, it is tempting to hypothesize that mitochondrial abnormality may contribute to early pathological changes in AD and ultimately to the characteristic cognitive impairments. Indeed, in the 3xTg mouse model of AD, mitochondrial bioenergy deficiency appears earlier than the pathological features of AD such as abnormal cognitive behavior, NFTs, and Aβ (Yao et al., 2009). Similarly, a mitochondrial abnormality is one of the earliest and most prominent features in the hippocampus of AD patients, occurring even before the appearance of Aβ and NFTs (Manczak et al., 2004). Whether estrogen deficiency leads to mitochondrial defects that precede and, therefore, help cause later AD pathological changes in postmenopausal women is unclear.

Epidemiological studies have shown that two-thirds of AD patients are women, which has been attributed to their longer average lifespan (Farrer et al., 1997). Increasing evidence shows that estrogen deficiency after menopause is associated with an increased risk of AD (Paganini-Hill and Henderson, 1994; Yue et al., 2005; Yao et al., 2012; Villa et al., 2016). Ovariectomy of premenopausal women increases the risk of AD by 40% (Rocca et al., 2010). The average woman experiences menopause around age 50 (Zhu et al., 2019), and the average age of AD onset in females is around 80 years (Beam et al., 2018). Several studies have demonstrated that estrogen replacement therapy can reduce or delay the onset of AD (Tang et al., 1996; Yaffe et al., 1998; van Duijn, 1999). A recent multimodal brain imaging study showed that a decrease in circulating estrogen is the main risk factor for female-specific brain abnormalities in AD (Rahman et al., 2020).

How estrogen deficiency leads to AD remains unclear, and it may involve perturbation of mitochondrial activity. Many estrogen-regulated signaling pathways are concentrated in mitochondria (Nilsen and Diaz Brinton, 2003; Brinton, 2008). Ovarian-produced 17β-estradiol (E2) is the most commonly circulating estrogen in women and can increase the activity and expression of several mitochondrial proteins involved in cellular respiration by acting on estrogen receptors in the mitochondria, namely complex Iβ subunit 8, complex IV, and complex V (Nilsen et al., 2007; Irwin et al., 2012). E2 stimulates the skeletal muscle to promote mitochondrial biogenesis, proliferation, and oxidative capacity (Capllonch-Amer et al., 2014). In the 3xTg AD mouse model, E2 can promote mitochondrial biogenesis, prevent free radical damage, and upregulate Aβ-degrading enzymes to reduce Aβ deposition (Brinton, 2009; Simpkins et al., 2010; Zhao et al., 2011). The function of estrogen is usually regulated by nuclear receptors, estrogen receptor alpha (ERα), and estrogen receptor beta (ERβ; Katzenellenbogen, 1996; Veenman, 2020), as well as membrane receptors, G protein-binding estrogen receptor-1 (GPER-1; Liu et al., 2009). ERα and ERβ are widely distributed in the central nervous system (Pérez et al., 2003). Many studies have found the presence of mitochondrial estrogen receptors (Yager and Chen, 2007; Mitterling et al., 2010; Irwin et al., 2012). ERα and ERβ were shown to directly bind mitochondrial DNA *in vitro* through mitochondrial estrogen response elements, and the binding response was increased with exposure to E2 (Chen et al., 2004). Estrogen through ERβ-mediated regulation of mtDNA transcription to regulate mitochondrial function (Yang et al., 2004; Irwin et al., 2012).

Using a postmenopausal mouse model, we explored here whether and how estrogen deficiency affects mitochondria, and whether this, in turn, relates to neuronal damage and cognitive impairment. Understanding the estrogen-dependent role of dynamic changes of hippocampal mitochondria in postmenopausal AD pathology will develop targeted drugs to better prevent or delay the occurrence of AD.

## Materials and Methods

### Animals

Seventy-two adult female C57BL/6J mice at 3 months of age, weighing 20–25 g (Shandong Skobas Biotechnology, Jinan, China) were used in the experiments. Animals were housed in plastic cages with controlled temperature (24 ± 2°C) and humidity (40–50%), and kept on a 12-h light/dark cycle. Mice had free access to food and water. Experimental protocols were approved by the Committee of Animal Experimental Ethics of Shandong First Medical University and conducted in accordance with the US National Institutes of Health “Guide for the Care and Use of Laboratory Animals.”

### Experimental Design

Mice were randomly divided into two groups (*n* = 36 per group): sham operation (Sham) and ovariectomy operation (OVX). After a 7-day adaptation period, sham operation or bilateral ovariectomy was performed under anesthesia with 0.3% sodium pentobarbital (0.1 ml/10 g) administered by intraperitoneal (IP) injection. A longitudinal incision was made inferior to the rib cage on the dorsolateral body wall. Mice subjected to the sham surgical procedures had a piece of fat excised from the body wall. In the OVX group, the bilateral ovaries were exteriorized, ligated, and excised. Eight mice from each group were sacrificed at 2 weeks, 1, 2, and 3 months after surgery.

Behavioral testing was performed 6 days before mice were sacrificed. After sacrifice, blood samples were collected from the orbital sinus, and serum estradiol was measured by enzyme-linked immunosorbent assay (ELISA). Neurons were identified based on immunofluorescence of microtubule-associated protein 2 (MAP2) on sections of the hippocampus. Total protein was extracted from hippocampal tissue upon sacrifice and analyzed by immunoblotting to assess synapses based on synaptic protein postsynaptic density-95 (PSD95); mitochondrial function, based on NADH: ubiquinone oxidoreductase subunit B8 (NDUFB8), Succinate dehydrogenase B (SDHB), Ubiquinol-cytochrome c reductase core protein 2 (UQCRC2), Mitochondrion cytochrome c oxidase subunit 1 (MTCO1), and ATP synthetase F1 complex α subunit (ATP5A1); mitochondrial biogenesis, based on peroxisome proliferator-activated receptor-gamma coactivator-1 alpha (PGC-1α), nuclear respiratory factor 1 (NRF1), and mitochondrial transcription factor A (mtTFA); mitochondrial dynamics, based on dynamin-related protein 1 (Drp1), optic atrophy 1 (OPA1), and mitofusin 2 (Mfn2); and mitophagy, based on Phosphatase and Tensin Homolog -induced putative kinase 1 (PINK1) and microtubule-associated protein B (LC3B).

### Morris Water Maze (MWM) Test

The cognitive function of mice was evaluated by the Morris water maze (MWM) test as previously described (Bromley-Brits et al., 2011). Briefly, mice were trained by successively placing them in the water at a location equidistant from the target platform in each quadrant for 5 days. During each trial, the mouse was allowed 60 s to locate the target platform by itself. If it failed to find the platform within 60 s, the experimenter placed the mouse on the platform for 10 s and the time required to reach the platform (escape latency) was recorded as 60 s. On day 6, the target platform was removed and the time spent by the mouse in the target quadrant where the platform had been and the number of times the mice crossed into the target quadrant was recorded for 60 s. In order to exclude variations caused by the circadian rhythm, animals were trained and tested each day between 10:00 AM and 5:00 PM. The tracking information was processed by the Topscan Package (Clever Sys Inc.).

### Serum Estradiol ELISA

Blood samples were collected from the orbital sinus and serum was separated by centrifugation at 1,500× *g* for 15 min at 4°C. Serum estradiol was quantified using a mouse E2 ELISA kit (ml063198, Shanghai Enzyme-linked Biotechnology, Shanghai, China) according to the manufacturer’s instructions. Absorbance at 450 nm was recorded with a multifunctional microplate reader (TECAN, Switzerland).

### Immunofluorescence Analysis

Whole brains were collected, post-fixed in buffered 4% paraformaldehyde overnight at 4°C, dehydrated in a graded ethanol series, and embedded in paraffin. The tissue was sectioned to a thickness of 4 μm and then mounted on glass slides. The sections were dried in an oven at 60°C and stored at room temperature. The sections were deparaffinized, rehydrated, and subjected to antigen retrieval, then rinsed in distilled water. The sections were blocked in 3% bovine serum albumin (BSA; G5001, Servicebio, Wuhan, China) for 30 min, and then incubated with anti-MAP2 antibody (GB11128-2, 1:200, Servicebio) overnight at 4°C. The next morning, the sections were washed in distilled water and incubated with horseradish peroxidase-conjugated goat anti-rabbit secondary antibody (GB23303, 1:200, Servicebio) for 1 h in darkness at room temperature, then incubated with 4′,6-diamidino-2-phenylindole (DAPI, G1012, Servicebio) for 10 min. The sections were washed in phosphate-buffered saline (PBS), incubated with spontaneous fluorescence quenching reagent (GB1221, Servicebio) for 5 min, washed again in PBS, and mounted on microscope slides with an anti-fade mounting medium. Sections were observed under a fluorescence microscope (Axioscope 5, Carl Zeiss, Jena, Germany), and images were captured with a slice scanner (Pannoramic MIDI, Danjier, Jjinan, China) and processed with Image-Pro Plus software version 6.0 (Media Cybernetics Corp., Bethesda, MD).

### Immunoblotting Analysis

Proteins were isolated from brain tissue and analyzed *via* Western blotting by standard methods (Zhou et al., 2021). Briefly, the hippocampus was homogenized in the presence of protease and phosphatase inhibitors (P1206, Solarbio, Shanghai, China), then centrifuged at 14,500× *g* for 10 min at 4°C. The supernatants were collected and the total protein concentration was measured by BCA assay (PC0020, Solarbio). Samples were run on a 10 or 12% SDS-PAGE gel and transferred to a polyvinylidene fluoride (PVDF) membrane (IPVH00010, Merck Millipore, Darmstadt, Germany). Membranes were blocked in 5% milk for 2 h at room temperature and stained sequentially for proteins of interest. For each protein, membranes were incubated with a primary antibody overnight at 4°C, washed in tris buffered saline with 0.1% Tween 20, and incubated with the relevant secondary antibody for 1 h at room temperature before a final wash.

Primary antibodies were against the following proteins: PSD95 (ab18258, 1:1,000, Abcam, Cambridge, UK), NDUFB8 (ab192878, 1:4,000, Abcam), SDHB (ab14714, 1:1,000, Abcam), UQCRC2 (ab203832, 1:1,000, Abcam), MTCO1 (ab203912, 1:2,000, Abcam), ATP5A1 (ab14748, 1:1,000, Abcam), PGC-1α (ab54481, 1:2,000, Abcam), NRF1 (46743, 1:1,000, Cell Signaling Technology, Danvers, MA, USA), mtTFA (ab252432, 1:2,000, Abcam), Mfn2 (ab56889, 1:1,000, Abcam), OPA1 (ab157457, 1:1,000, Abcam), Drp1 (ab184247, 1:1,000, Abcam), p-Drp1 (ser 637; ab193216, 1:1,000, Abcam), LC3B (ab192890, 1:1,000, Abcam), PINK1(ab23707, 1:1,000, Abcam), β-actin (TA-09, 1:2,000, Zhongshan Golden Bridge, Beijing, China), and GAPDH (TA-08, 1:2,000, Zhongshan Golden Bridge). The horseradish peroxidase-conjugated secondary antibodies were goat anti-mouse (ZB-2305, 1:3,000, Zhongshan Golden Bridge) and goat anti-rabbit (ZB-2301, 1:3,000, Zhongshan Golden Bridge).

Immunoreactivity was visualized using Enhanced Chemiluminescence reagents (BL520A, Biosharp, Beijing, China) according to the manufacturer’s instructions, and images were acquired with the AI-600 System (GE, USA). Densitometry was performed using the Image-Pro Plus software version 6.0 and proteins of interest were normalized to GAPDH or β-actin. Normalized protein levels were expressed relative to those in the 2 weeks Sham group.

### Statistical Analysis

All data were presented as mean ± standard error of the mean (SEM) and analyzed using GraphPad Prism version 8 (GraphPad Software, San Diego, CA, USA). Before the significance test, the Shapiro-Wilk test is used to verify the normal distribution, and all data are consistent with the normal distribution. Data were analyzed with two-way analysis of variance (ANOVA), followed by Dunnett or Bonferroni *post hoc* tests for pairwise comparison. Statistical significance was set at *p* < 0.05.

## Results

### Estradiol Levels in Ovariectomized Mice

**Figure 1:**
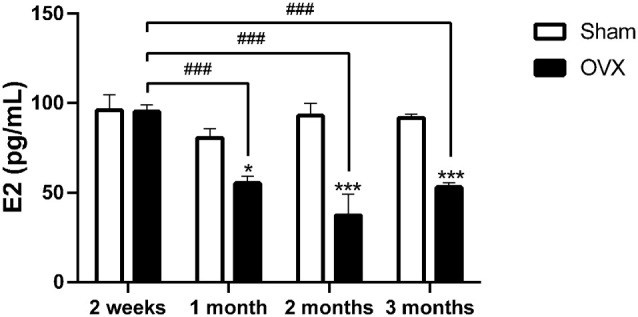
Serum estradiol (E2) level in mice after sham surgery (Sham) or ovariectomy (OVX). Bars represent mean values ± standard error of the mean (SEM). *p<0.05, ***p<0.001 compared with the Sham mice at the same timepoint; ^###^*p*<0.001 compared with the 2 weeks OVX mice (*n* = 6).

At 2 weeks after the surgery, serum E2 levels in OVX mice were not significantly different from those in the Sham mice. However, OVX mice exhibited significantly lower serum E2 levels than the Sham mice at 1 (*p* < 0.05), 2 (*p* < 0.001), and 3 months (*p* < 0.001) after surgery. Serum E2 levels in the OVX mice were significantly lower at 1, 2, and 3 months than at 2 weeks (all *p* < 0.001; Figure 1).

### Estrogen Deficiency Contributes to Cognitive Impairment

**Figure 2:**
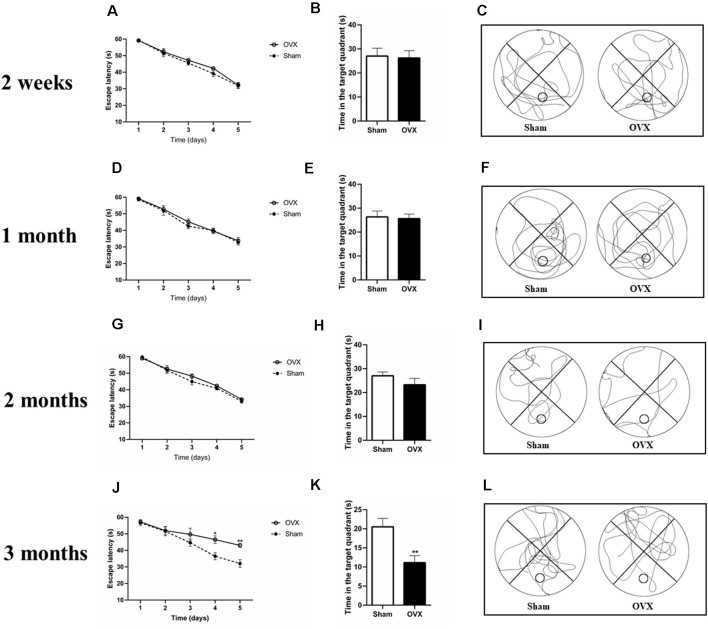
Spatial cognitive impairments in ovariectomized mice (OVX) as measured in the Morris water maze test. **(A,D,G,J)** Escape latency. **(B,E,H,K)** Time spent in the target quadrant. **(C,F,I,L)** Representative animal trajectories during 60 s on day six. The circle in a lower quadrant marks the original placement on the platform. Bars represent mean values ± standard error of the mean (SEM). *p < 0.05, **p < 0.01 compared with the Sham group at the same timepoint (*n* = 8).

At 2 weeks, 1, and 2 months after surgery, the Sham and OVX mice showed a similar reduction in escape latency during MWM training (Figures 2A,D,G). In the trial test on day 6 (with the platform removed), the OVX mice spent a similar amount of time in the target quadrant as the Sham mice at 2 weeks, 1, and 2 months after surgery (Figures 2B,C,E,F,H,I).

At 3 months after surgery, the OVX and Sham mice showed a reduced escape latency during the training process up to day 4 (Figure 2J, *p* < 0.05). On day 5, the OVX mice showed longer escape latency than the Sham mice (*p* < 0.01). Similarly, in the trial test (with the platform removed), the Sham mice spent significantly more time in the target quadrant than the OVX mice (Figures 2K,L, *p* < 0.01).

### Effects of Estrogen Deficiency on Hippocampal Neurons and Synapses

**Figure 3:**
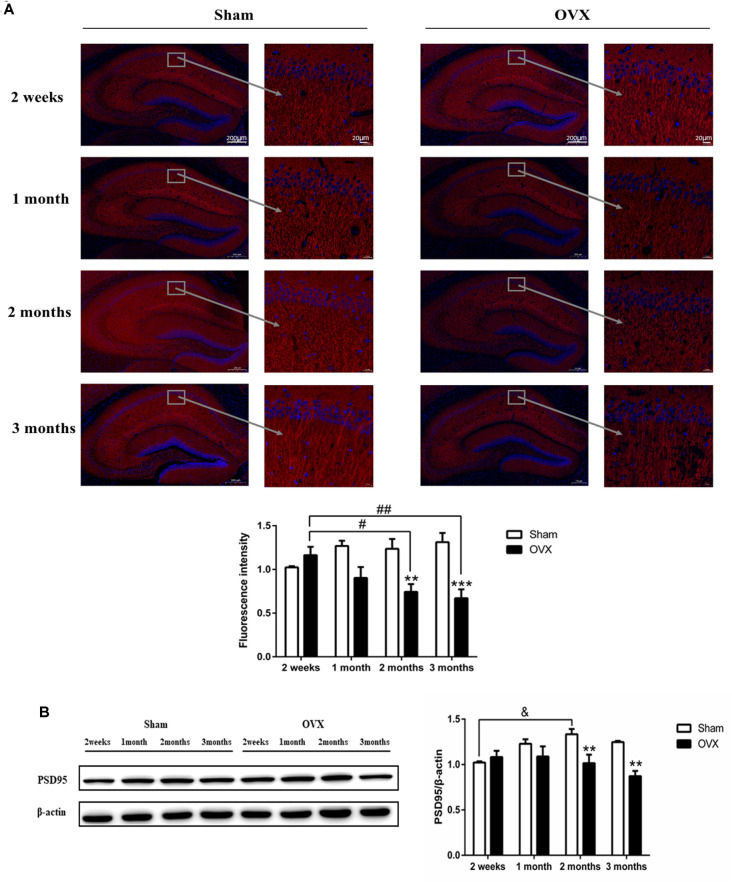
The neuronal structure in the hippocampus CA1 region is damaged in response to ovariectomy (OVX). **(A)** MAP2 immunofluorescence and quantitation. **(B)** Immunoblotting analysis of PSD95 and quantitation. Bars represent mean values ± standard error of the mean (SEM). **p < 0.01, ***p < 0.001 compared with the Sham mice at the same timepoint; ^&^*p* < 0.05 compared with the 2-week Sham mice; ^#^*p* < 0.05, ^##^*p* < 0.01 compared with the 2 weeks OVX mice (*n* = 4).

MAP2 is considered an important component of the cytoskeleton of neurons (Sánchez et al., 2000). In the hippocampal CA1 region, the expression of MAP2 in the OVX mice was significantly reduced compared with the Sham mice of the same age 2 months after surgery (*p* < 0.01), which persisted to 3 months (*p* < 0.001; Figure 3A). Within the OVX mice, MAP2 expression decreased significantly and continuously from 2 weeks until 3 months after surgery and was significantly lower at 2 (*p* < 0.05) and 3 months (*p* < 0.01) than at 2 weeks after surgery.

PSD95 is a scaffold protein related to the assembly and function of the postsynaptic compact complex (Cao et al., 2005). Expression of PSD95 was significantly lower in the OVX mice than in the Sham mice of the same age at 2 (*p* < 0.01) and 3 months (*P* < 0.01) after surgery (Figure 3B). In fact, PSD95 expression within OVX mice continuously decreased from 2 weeks to 2 months after surgery, whereas expression in the Sham mice increased or remained constant over the same period and was significantly increased at 2 months (*p* < 0.05) than at 2 weeks after surgery.

### Effect of Estrogen Deficiency on Mitochondrial Function in Hippocampus

**Figure 4:**
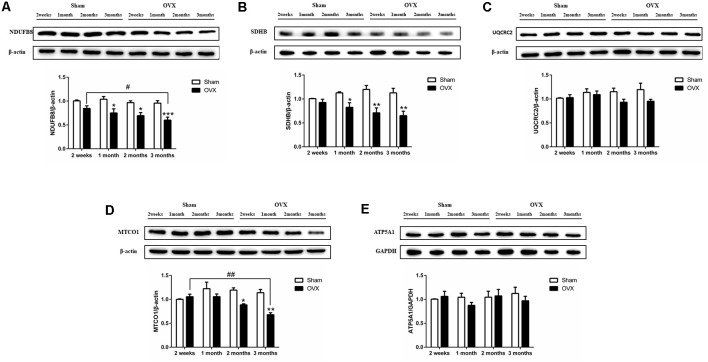
Levels of the mitochondrial function proteins **(A)** NDUFB8, **(B)** SDHB, **(C)** UQCRC2, **(D)** MTCO1, **(E)** ATP5A1 in the hippocampus of sham-operated (Sham) and ovariectomized (OVX) mice. Bars represent mean values ± standard error of the mean (SEM). *p < 0.05 **p < 0.01, ***p < 0.001 compared with the Sham mice at the same timepoint; ^#^*p* < 0.05, ^##^*p* < 0.01 compared with the 2 weeks OVX mice (*n* = 4).

Mitochondrial function was assessed by measuring the protein expression of mitochondrial respiratory chain enzymes complex I (NDUFB8), complex II (SDHB), complex III (UQCRC2), complex IV (MTCO1), and complex V (ATP5A1) in the hippocampus. Compared to the Sham mice, the OVX mice showed significantly lower levels of NDUFB8 (Figure 4A), SDHB (Figure 4B) and MTCO1 (Figure 4D) at 1 (*p* < 0.05 for NDUFB8 and SDHB), 2 (*p* < 0.05; *p* < 0.01; *p* < 0.05), and 3 months (*p* < 0.001; *p* < 0.01; *p* < 0.01). NDUFB8 and MTCO1 levels in the OVX mice continuously decreased from 2 weeks to 3 months and were significantly lower at 3 months (*p* < 0.05; *p* < 0.01) than at 2 weeks after surgery. In contrast, UQCRC2 and ATP5A1 levels did not differ significantly between the OVX and Sham mice at any timepoint, nor did levels vary significantly within either group over time (Figures 4C–E).

### Effect of Estrogen Deficiency on Mitochondrial Biogenesis in Hippocampus

**Figure 5:**
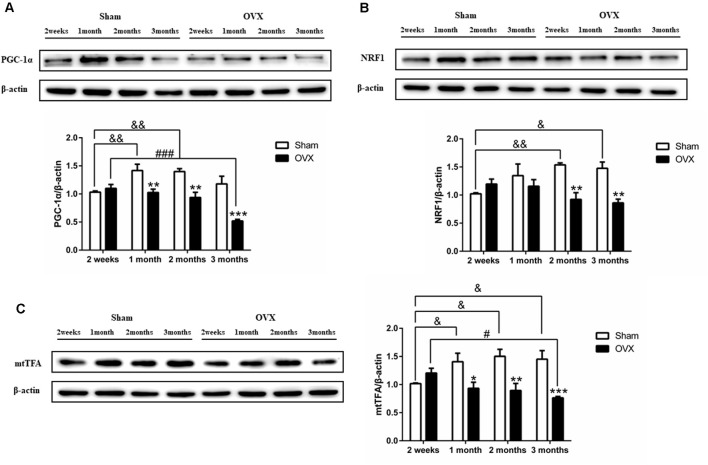
Levels of the mitochondrial biogenesis proteins **(A)** PGC-1α, **(B)** NRF1, and **(C)** mtTFA in the hippocampus of sham-operated (Sham) and ovariectomized (OVX) mice. Bars represent mean values ± standard error of the mean (SEM). *p < 0.05, **p < 0.01, ***p < 0.001 compared with the Sham mice at the same timepoint; ^&^*p* < 0.05, ^&&^*p* < 0.01 compared with the 2 weeks Sham mice; ^#^*p* < 0.05, ^###^*p* < 0.001 compared with the 2-week OVX group (*n* = 4).

PGC-1α is considered the master regulator of mitochondrial biogenesis (Wu et al., 1999). The expression of PGC-1α was significantly lower in the OVX mice than in the Sham mice at 1 (*p* < 0.01), 2 (*p* < 0.01), and 3 months (*p* < 0.001) after surgery (Figure 5A). In fact, levels continuously decreased in the OVX mice from 2 weeks to 3 months and were significantly lower at 3 months (*p* < 0.001) than at 2 weeks after surgery, whereas levels increased in the Sham mice from 2 weeks to 1 month and were significantly increased at 1 (*p* < 0.01) and 2 months (*p* < 0.01) than at 2 weeks after surgery.

PGC-1α regulates mitochondrial biogenesis by activating multiple transcription factors, including NRF1 and mtTFA (Wu et al., 1999). Compared to the Sham mice, the OVX mice showed significantly lower levels of NRF1 (Figure 5B) and mtTFA (Figure 5C) at 1 (*p* < 0.05 for mtTFA), 2 (*p* < 0.01; *P* < 0.01), and 3 months (*p* < 0.01; *p* < 0.001). The decrease in mtTFA expression in OVX mice mirrored the PGC-1α trend (Figures 5A–C). NRF1 and mtTFA levels in the OVX mice continuously decreased from 2 weeks to 3 months and was significantly lower at 3 months (*p* < 0.05 for mtTFA) than at 2 weeks after surgery, whereas the levels in the Sham mice increased or remained constant over the same period and was significantly increased at 1 (*p* < 0.05 for mtTFA), 2 (*p* < 0.01; *p* < 0.05) and 3 months (*p* < 0.05; *p* < 0.05) than at 2 weeks after surgery.

### Effect of Estrogen Deficiency on Mitochondrial Dynamics in Hippocampus

**Figure 6:**
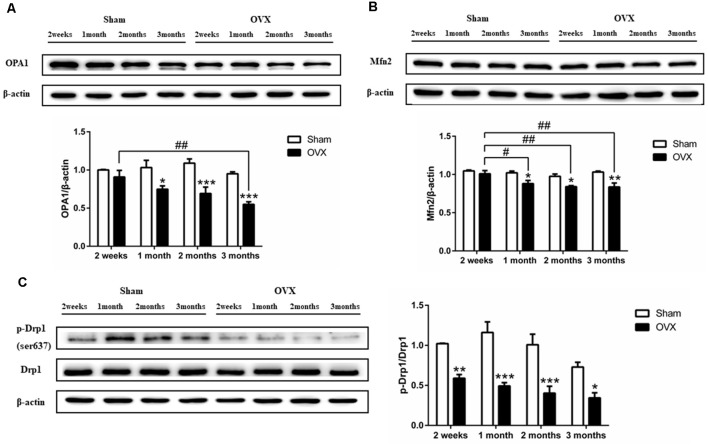
Levels of the mitochondrial dynamics proteins **(A)** OPA1, **(B)** Mfn2 **(B)**, and **(C)** phosphorylated Drp1 (p-Drp1) in the hippocampus of mice after sham surgery (Sham) or ovariectomy (OVX). Bars represent mean values ± standard error of the mean (SEM). *p < 0.05, **p < 0.01, ***p < 0.001 compared with the Sham mice at the same timepoint; ^#^*p* < 0.05, ^##^*p* < 0.01 compared with the 2-week OVX group (*n* = 4).

Levels of the mitochondrial fusion proteins OPA1 (Figure 6A) and Mfn2 (Figure 6B) were significantly lower in the hippocampus of the OVX mice than in the Sham mice at 1 (all *p* < 0.05), 2 (*p* < 0.001 for OPA1; *p* < 0.05 for Mfn2), and 3 months after surgery (*p* < 0.001 for OPA1; *p* < 0.01 for Mfn2). OPA1 and Mfn2 expression decreased over time in the OVX mice, with the level at 1 (*p* < 0.05 for Mfn2), 2 (*p* < 0.01 for Mfn2), and 3 months (*p* < 0.01; *p* < 0.01) significantly lower than the level at 2 weeks. Levels of Drp1 phosphorylation at Ser637 were significantly lower in the OVX mice than in the Sham mice at all timepoints (Figure 6C).

### Effects of Estrogen Deficiency on Hippocampal Mitophagy

**Figure 7:**
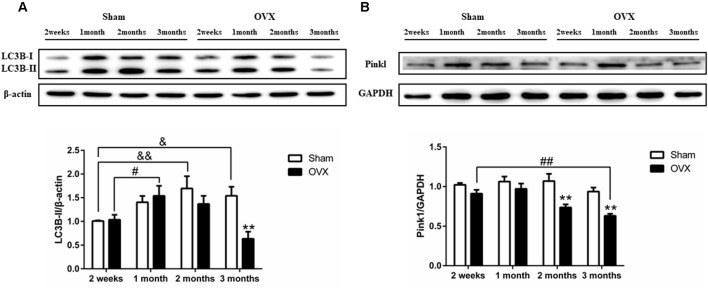
Levels of the autophagy and mitophagy proteins **(A)** LC3B-II and **(B)** Pink1 in the hippocampus of mice after sham surgery (Sham) or ovariectomy (OVX). Bars represent mean values ± standard error of the mean (SEM). **p < 0.01 compared with the Sham mice at the same timepoint; ^ &^*p* < 0.05, ^&&^*p* < 0.01 compared with the 2 weeks Sham mice; ^ #^*p* < 0.05, ^##^*p* < 0.01 compared with the 2 weeks OVX mice (*n* = 4).

Levels of LC3B-II were initially higher in the OVX mice than in the Sham mice, then they gradually became lower than in the Sham mice at later times, with the difference becoming significant at 3 months after surgery (Figure 7A, *p* < 0.01). Pink1 expression (Figure 7B) was significantly lower in the OVX mice than in the Sham mice at 2 months after surgery (*p* < 0.01), which persisted to 3 months (*p* < 0.01). Within the OVX mice, LC3B-II protein expression was significantly increased at 1 month than at 2 weeks after surgery (*p* < 0.05) and continuously decreased from 1 month to 3 months. Pink1 levels in the OVX mice continuously decreased from 1 month to 3 months and were significantly lower at 3 months (*p* < 0.01) than at 2 weeks after surgery.

## Discussion

Sex differences in neurodegenerative diseases suggest that sex hormones may play an important role regardless of age (Zárate et al., 2017; Scheyer et al., 2018). For the purpose of understanding the dynamic changes in the brain and clarifying the etiology of estrogen-related AD, we surgically removed the ovaries of mice to imitate menopause. Behavioral and histopathological observations at 2 weeks, 1, 2, and 3 months after ovariectomy allowed us to characterize the progression of AD in our postmenopausal mouse model. We can better understand the dynamic changes of hippocampal mitochondria after estrogen deficiency.

We found that the serum E2 levels of ovariectomized mice did not change significantly in the first 2 weeks, but they significantly decreased at all later timepoints (Figure 1). This may be related to stress compensation of estrogen levels in the short–term after ovariectomy. Ovariectomy can induce androstenedione and androstanol to produce estrogen by aromatase in adipose tissue, but this stress-dependent effect is only temporary, and estrogen levels later decrease (Aiman et al., 1978; Nelson and Bulun, 2001).

Ovariectomized young rodents with chronic estrogen deficiency show deficits in spatial learning tasks, including radial arm mazes and MWMs (Heikkinen et al., 2004; Gibbs et al., 2011). Consistent with previous studies, our OVX mice showed significantly lower memory and problem-solving ability in the MWM test than Sham animals at 3 months after surgery (Figures 2K–M). Postmenopausal women receiving estrogen replacement therapy may have improved cognitive function (Duarte et al., 2016; Merlo et al., 2017). Estrogen prevents aberrant hippocampal neuronal and cognitive deficits (Sales et al., 2010; Yazğan and Nazıroğlu, 2017).

Synapses are formed by connections between two neurons, allowing one neuron to transmit signals to another. Synaptic abnormalities may be related to a variety of nervous system diseases (Fu and Ip, 2017). PSD95 plays an important role in the formation and maturation of excitatory synapses during the development of hippocampal neurons (Gerrow et al., 2006; Bustos et al., 2017). MAP2 is a mature neuron marker, located in the cytoskeleton of neurons and plays an important role in the growth of dendrites (Heidemann, 1996). Our data showed that the hippocampal expression of PSD95 and MAP2 decreased 2 months after ovariectomy, with these decreases persisting until at least 3 months. The study found that the spines and synaptic boutons in the hippocampal CA1 area of ovariectomized rats were reduced, which was reversed after estrogen treatment (Woolley and McEwen, 1992). Consistent with our results, estrogen deficiency can lead to the loss of synapses.

Mitochondrial dysfunction is involved in the pathological process of neurodegenerative diseases (Schon and Przedborski, 2011). Estradiol participates in the regulation of the activity and expression of brain mitochondrial respiratory chain enzymes (Nilsen et al., 2007; Irwin et al., 2012). Our data showed that 1 month after ovariectomy, the expression of hippocampal mitochondrial complex proteins NDUFB8 and SDHB decreased and 2 months after ovariectomy, the expression of hippocampal MTCO1 decreased. Decreased expression of mitochondrial oxidative phosphorylation protein could reduce the mitochondrial metabolism efficiency and trigger a series of dynamic changes in mitochondria. In this study, we further investigated the impact of E2 deficiency on mitochondrial bioenergetics, dynamics, and mitophagy over time. Our results suggest that ovarian hormone deficiency induced by ovariectomy causes a significant decrease in mitochondrial function in the hippocampus before cognitive impairment. Specifically, the hippocampus of OVX mice showed decreased mitochondrial biogenesis, mitophagy, and mitochondrial fusion, as well as an increase in mitochondrial fission. These data support the hypothesis that ovarian hormone deficiency compromises mitochondrial function (Yao et al., 2012).

Mitochondrial biogenesis is the process of the formation of new mitochondria by the growth and division of pre-existing mitochondria, which increases the mitochondrial mass in cells (Li et al., 2017). PGC-1α is a major regulator of mitochondrial biogenesis. It interacts with several DNA-binding transcription factors to regulate mitochondrial biogenesis and dynamic changes, thus maintaining mitochondrial pool (Wu et al., 1999; Dorn et al., 2015). Levels of mRNAs encoding PGC-1α, NRF1, and mtTFA are decreased in the brains of 1-month-old 3xTg AD mice (Singulani et al., 2020). The Swedish mutation (APPswe) reduces the expression of PGC-1α and impairs mitochondrial biogenesis in cellular models of AD (Sheng et al., 2012). Our data showed that the hippocampal expression of PGC-1α and mtTFA decreased 1 month after ovariectomy, and the hippocampal expression of NRF1 decreased 2 months after surgery, with these decreases persisting until at least 3 months (Figure 5). Since estrogen deficiency leads to a mitochondrial biogenesis defect, overexpression of PGC-1α to increase mitochondrial biogenesis is a potential strategy to treat mitochondrial diseases (Srivastava et al., 2009). PGC-1α can interact with other transcription factors, such as estrogen receptors, peroxisome proliferator-activated receptors (PPARs), and antioxidant proteins (Ventura-Clapier et al., 2008). This study found that estrogen deficiency can lead to mitochondrial biogenesis damage 1 month after ovariectomy. And considering that mitochondrial biogenesis is an early symptom of AD, and PGC-1α interacts with estrogen receptors. It was attractive to investigate estrogen regulate PGC-1α for improving brain mitochondria biogenesis in postmenopausal women with AD.

Mitochondria are highly dynamic organelles that undergo continuous fusion and fission in the cytoplasm to maintain the mitochondrial pool (Zhu et al., 2013). OPA1 on the inner mitochondrial membrane and Mfn2 on the outer mitochondrial membrane regulates mitochondrial fusion. At 1 month after ovariectomy, we found that OPA1 and Mfn2 were significantly decreased, indicating impaired mitochondrial fusion and, therefore, an increased mitochondrial pool. Drp1 regulates the fission of mitochondria, and its phosphorylation at Ser637 inhibits fission (Knott et al., 2008). Our data show that at 2 weeks after ovariectomy, levels of phosphorylated Drp1 (Ser637) significantly increased, indicating impaired fission and a smaller mitochondrial pool. Thus, by 1 month after ovariectomy, estrogen deficiency leads to an imbalance of fission and fusion, causing mitochondrial fragmentation, which has been linked to nervous system disorders (Mishra and Chan, 2014). Studies have found that estrogen deficiency can damage the mitochondrial dynamics of the heart and skeletal muscle, which can be reversed by estrogen replacement therapy (Capllonch-Amer et al., 2014; Garvin et al., 2017; Minta et al., 2018). Our study extends the literature by providing evidence that mitochondrial dysregulation triggered by estrogen deficiency contributes to cognitive impairment in AD.

Impaired mitophagy may be an important cause of AD in menopausal women. Mitophagy refers to the selective removal of mitochondria through the autophagy mechanism, which is one way that the cell degrades dysfunctional mitochondria. Previous studies have found that estrogen promotes mitochondrial autophagy in a number of diseases, including Osteoarthritis and myocardial ischemia/reperfusion injury (Feng et al., 2017; Fan et al., 2018; Sun et al., 2018; Mei et al., 2020). Defective mitophagy contributes to the pathology of neural degeneration since it prevents the destruction of damaged mitochondria (Ni et al., 2015; Franco-Iborra et al., 2018). Damage to mitochondria can alter the mitochondrial membrane potential, activating Pink1 in the outer mitochondrial membrane, which initiates autophagy (Nguyen et al., 2016). In mouse models of AD, the decrease of Pink can aggravate synapse loss and cognitive dysfunction (Rodríguez-Navarro et al., 2008), which can be mitigated by promoting mitophagy (Du et al., 2017; Fang et al., 2019). Our data show that hippocampal mitophagy in mice was inhibited from 2 months after ovariectomy through at least 3 months. Our results imply that estrogen replacement therapy in early postmenopausal women may be a method to preserve mitophagy and slow the progression of AD.

Estrogen replacement therapy (ERT) can effectively reduce the cognitive impairment of some but not all postmenopausal women (Espeland et al., 2004; Vedder et al., 2014). Many evidence show that the effectiveness of ERT depends on its application in a critical period (Zandi et al., 2002; Qin et al., 2020). However, large clinical trials have indicated that HRT increases the risk of breast cancer and stroke (Beral, 2003; Stahlberg et al., 2004; Henderson and Lobo, 2012). Selective estrogen receptor modulators (SERM) by selectively affecting certain types of estrogen receptors as partial agonists, also acts as an antagonist of other types of signaling systems related to natural estrogen (Jenkins et al., 2021). SERM has the advantage of reducing the risk of estrogen-dependent tumors (Coman et al., 2017). Considering that estrogen regulates mitochondrial bioenergetics by regulating the transcription of mitochondrial DNA through estrogen receptors located on the mitochondria (Rettberg et al., 2014). Our study found that estrogen deficiency can lead to a series of mitochondrial damage and then affect cognitive function, which provides an important insight for SERMs as a treatment for cognitive and neurodegenerative diseases.

Understanding the impact of estrogen deficiency on the brain can further advance our understanding of AD pathogenesis in postmenopausal women. Our study in ovariectomized mice suggests that disruption of mitochondria function precedes cognitive impairment, which may help explain the subsequent damage to hippocampal neurons, synapses, and cognitive function that is characteristic of AD. This may have interesting implications for treating or preventing AD symptoms in women.

## Conclusions

Estrogen deficiency appears to contribute to the cognitive defects of AD, through a mechanism that may involve disruption of mitochondrial biogenesis, fusion/fission, and function. These findings in mice justify further work to explore the possibility of improving mitochondrial function in postmenopausal women in order to delay or prevent AD symptoms.

## Data Availability Statement

The original contributions presented in the study are included in the article, further inquiries can be directed to the corresponding author/s.

## Ethics Statement

The animal study was reviewed and approved by the Committee of Animal Experimental Ethics of Shandong First Medical University.

## Author Contributions

WZ: conceptualization and writing. YH, XS, and LW: methodology and data curation. FZ and HZ: investigation. HY and YZ: conceptualization and writing. All authors contributed to the article and approved the submitted version.

## Conflict of Interest

The authors declare that the research was conducted in the absence of any commercial or financial relationships that could be construed as a potential conflict of interest.
